# Breathing Right… or Left! The Effects of Unilateral Nostril Breathing on Psychological and Cognitive Wellbeing: A Pilot Study

**DOI:** 10.3390/brainsci14040302

**Published:** 2024-03-23

**Authors:** Maria Elide Vanutelli, Chiara Grigis, Claudio Lucchiari

**Affiliations:** 1 Department of Philosophy “Piero Martinetti”, Università degli Studi di Milano, 20122 Milan, Italy; maria.vanutelli@unimib.it (M.E.V.); grigischiara@yahoo.it (C.G.); 2 Department of Psychology, University of Milano-Bicocca, 20126 Milan, Italy

**Keywords:** unilateral nostril breathing, pranayama, brain lateralization, psychological wellbeing, mind wandering, yoga

## Abstract

The impact of controlled breathing on cognitive and affective processing has been recognized since ancient times, giving rise to multiple practices aimed at achieving different psychophysical states, mostly related to mental clarity and focus, stress reduction, and relaxation. Previous scientific research explored the effects of forced unilateral nostril breathing (UNB) on brain activity and emotional and cognitive functions. Some evidence concluded that it had a contralateral effect, while other studies presented controversial results, making it difficult to come to an unambiguous interpretation. Also, a few studies specifically addressed wellbeing. In the present study, we invited a pilot sample of 20 participants to take part in an 8-day training program for breathing, and each person was assigned to either a unilateral right nostril (URNB) or left nostril breathing condition (ULNB). Then, each day, we assessed the participants’ wellbeing indices using their moods and mind wandering scales. The results revealed that, after the daily practice, both groups reported improved wellbeing perception. However, the effect was specifically related to the nostril involved. URNB produced more benefits in terms of stress reduction and relaxation, while ULNB significantly and increasingly reduced mind-wandering occurrences over time. Our results suggest that UNB can be effectively used to increase wellbeing in the general population. Additionally, they support the idea that understanding the effects of unilateral breathing on wellbeing and cognition requires a complex interpretive model with multiple brain networks to address bottom-up and top-down processes.

## 1. Introduction

### 1.1. Symmetry and Asymmetry in the Human Body

A characteristic feature of biological systems is their symmetrical organization, particularly on the left–right (L-R) axis. Throughout history, the quest to achieve balance between symmetry and asymmetry has widely inspired humankind, with its greatest expression being found in ancient Greece, where symmetry was extended from a pure geometric property to an aesthetic value applicable to art, architecture, music, and science. However, in nature, symmetry typically arises in biological structures with low complexity. When it comes to very complex organisms, it is easier to find asymmetrical functional patterns [1]. The brain is probably one of the most interesting examples, since the two hemispheres are not mirrored in function and structure [2,3]. They present a specialized organization of sensory, motor, and cognitive domains. Language [4,5], face perception [6], and emotion processing [7,8] are among the most studied domains in the field of hemispheric differences. Hemispheric specialization provides many advantages in terms of efficiency since it allows tasks to be carried out in parallel and improves multitasking capabilities [1,9,10]; it also reduces the redundancy of processing units and finalizes action control [10].

In addition to cortical activity, other important vital functions for an organism appear to be asymmetrical, including respiration. Nasal airflow is not identical between the two nostrils since, at any given time, one nostril is dominant over the other, with a greater airflow from one side. This occurs because the erectile tissue causes a transient obstruction in the nasal passage, alternating between the two nostrils, with a periodicity called the nasal cycle. Thus, it can be defined as a rhythm of nasal congestion and decongestion that repeats approximately every 60–240 min [11,12].

Scientific evidence proved that the asymmetry of nasal airflow and brain asymmetry are linked. However, previous knowledge has already postulated this relationship.

### 1.2. The Influence of Breathing on Hemispheric Activity According to Yogic Tradition

According to the millennia-old yogic tradition, the body is crossed by numerous energy channels (*nadis*). The most important ones are *ida* and *pingala*, which run from the pubis to the top of the skull. They intersect each other along the vertical line of the body, which is occupied by another important channel, *sushumna*, from which *kundalini*, or vital energy, ascends. *Ida* and *pingala* are associated with a specific type of energy and are lateralized through the nostrils; *ida* is connected to the left nostril and right hemisphere. It represents lunar, feminine energy, which is calm, intuitive, and reflexive (directed inward) with cooling effects. *Pingala* is connected to the right nostril and left hemisphere, representing solar, masculine energy, which is stimulating, active, rational, and directed outward with heat-generating effects. The flows through these energy channels can be balanced or unbalanced with an activating or relaxing effect. Their balance changes based on the nasal cycle, but it can be modulated by directing breathing through *pranayama* techniques (from the Sanskrit word “*prana*”, meaning life energy, and “*yama*”, meaning control). In detail, *anuloma-viloma pranayama* can influence the activity of the cerebral hemispheres [13] and corresponding psychophysical states [14], and it can be carried out by voluntarily breathing through only one nostril and closing the other with the fingers of the dominant hand [15].

Thus, there are two variants, namely *surya anuloma-viloma* (*SAV*: “*surya*”, meaning the sun), in which both inhalation and exhalation occur only from the right nostril (URNB) without any retention, and *chandra anuloma-viloma* (*CAV*: “*chandra*”, meaning the moon), in which inhalation and exhalation occur only from the left nostril (ULNB). These two variations are thought to influence psychophysical wellbeing in different ways. In detail, *CAV* would directly control the *ida* channel with cooling, relaxing effects through the activation of the parasympathetic system, while SAV stimulates the *pingala* channel with warming, activating effects due to the engagement of the sympathetic system [16,17]. Also, *anuloma-viloma* is able to selectively stimulate the contralateral hemisphere, supporting its activity and improving its performance [14].

### 1.3. The Influence of Breathing on Hemispheric Activity According to Neuroscience

Several scientific studies have been conducted to understand whether and how airflow lateralization affects the cerebral hemispheres. Regarding autonomic activity, previous research findings seem to confirm the presence of a contralateral effect, as proposed by yogic tradition. Experiments conducted in the 1980s showed that the nasal cycle is regulated by the autonomic nervous system [18]. Since most autonomic nerve fibers are lateralized, the dominant nostril during the nasal cycle corresponds to sympathetic activation in the corresponding side of the body and central nervous system. In the brain, the increase in sympathetic tone produces ipsilateral cortical vasoconstriction with a consequent reduction in cognitive activity in that hemisphere. To compensate for the decrease in blood flow on one side, the other side undergoes vasodilation caused by an increase in parasympathetic tone [14]. The same mechanism applies in the case of forced breathing techniques, such as *anuloma-viloma pranayama*, which, in Western societies, is known as unilateral nostril breathing (UNB). Shannahoff-Khalsa reports numerous studies relating indices of sympathetic system activation to URNB, including increased plasma glucose levels and an increased heart rate [14]. In contrast, ULNB appears to be related to the stimulation of the parasympathetic system. For example, Telles and colleagues recorded a significant increase in the galvanic skin response (GSR) following breathing from the left nostril [16,19,20]. Because of its calming effects, ULNB has been effectively used to treat hypertensive patients with very interesting results [21]. In fact, after only 27 rounds of ULNB, all of the measured cardiovascular parameters (including heart rate, systolic pressure, and pulse pressure) were immediately improved.

Regarding central nervous system activity, on the other hand, the evidence becomes less concordant, and it is more complex to draw unambiguous conclusions about the processes involved. The majority of studies showed a contralateral effect with electrophysiological and neuroimaging data [11,12,22]. For example, Jella and Shannahoff-khalsa [23] found a double dissociation, with ULNB improving spatial performance and URNB improving verbal performance. Also, higher levels of oxygenated hemoglobin (oxyHb) concentrations in the contralateral frontal lobes have been recorded [24]. However, evidence of an ipsilateral [25] or an absent relationship [26,27] exists.

More recent perspectives have tried to overcome this dialectic by proposing more complex explanatory anatomo-functional models. For example, based on some previous evidence [24,28], Niazi and colleagues [11] put another hypothesis on the table suggesting that brain lateralization could occur after breathing; they not only considered the lateral plane (left-right), but also the longitudinal one (anterior-posterior). Moreover, they proposed to avoid using an overly simplistic view when discussing the results of breathing and brain activity, emphasizing the need to include brain networks in the discussion, especially the functional connectivity mechanisms that may occur [11].

### 1.4. The Aims and Hypotheses of the Present Research

There are still a lot of inconclusive or conflicting data regarding the psychological and cognitive effects of UNB. Furthermore, previous studies are mainly based on the analysis of asymmetries related to different cognitive tasks, and there is still much to be investigated about psychological and cognitive wellbeing.

The modulation of nasal airflow has been shown to induce many beneficial effects on our psychophysical wellbeing, preventing asthma, cardiovascular and gastrointestinal problems, hypertension, chronic pain, and inflammatory states [29,30], as well on our mental health, helping to treat anxiety, obsessive compulsive disorder, depression, post-traumatic stress disorder, panic attacks, and addictions [14]. Thus, it is used in many clinical and non-clinical contexts to promote social skills [29] and psychophysical wellbeing [30,31,32,33].

However, two wellbeing parameters are still poorly addressed in the literature: emotional states and mind wandering. Indeed, people who mind wander more frequently also seem to have worse psychological wellbeing and more negative affects [34,35], and this is especially true when mind wandering is unintentional [36]. Moreover, individuals with depressive symptomatology are more likely to have high rates of mind wandering [34] and tend to report lower levels of happiness [37].

Furthermore, even on a theoretical level, emotions and mind wandering are associated with previous research on hemispheric asymmetry, especially in prefrontal areas. Mind wandering is traditionally associated with the activation of the Default Mode Network (DMN; [38,39]) and, therefore, is opposed to the functioning of the prefrontal cognitive control system, which tend to suppress the DMN and mind wandering during demanding tasks [40,41]. Interestingly, some studies reported an association between DMN activation and emotions, with negative emotions more commonly being reported during mind-wandering experiences [37], especially when internal narratives are unintentional and not goal-oriented [42]. These findings suggest that spontaneous, automatic, and uninterested mind wandering leads to more negative emotions than purposed thinking processes.

Thus, exploring the relationship between UNB and these cognitive/emotional processes also allows for the investigation of the underlying neurophysiological circuits. For example, a previous study by Niazi et al. [11] hypothesized the presence of a connection betweenposterior structures and DMN during left nostril breathing.

Drawing on relevant studies in the literature, we can therefore propose the following hypotheses:

- Right nostril breathing activates the sympathetic nervous system, causing an increase in arousal and thus energizing emotional states (happiness), increasing cognitive focus, and decreasing mind wandering [7].
- Breathing from the left nostril, on the other hand, activates the parasympathetic nervous system more, producing a state of decreased arousal and activating the DMN, which, in turn, favors mind wandering [34,35].

To test the research hypotheses, we ran a study on a small pilot sample of 20 participants who took part in a UNB training program for 8 days while their mood states and mind-wandering behaviors were monitored.

## 2. Methods

### 2.1. Participants

Twenty adults, comprising 12 women and 8 men (M_age_ = 22.95; SD = 2.35), took part in this research. Considering the variables used to measure wellbeing and the pilot nature of this study, we chose our sample size in agreement with previous studies with a similar setting based on training and longitudinal assessments (e.g., [26]). Inclusion criteria were (1) being right-handed; (2) having normal or corrected-to-normal visual acuity; and (3) aged between 18 and 45 years old. Previous history of psychiatric or neurological disorders and previous long-term experience with breathing and meditation techniques were considered exclusion criteria.

By adopting the snowball sampling method, this study was advertised on the university website and major social channels. All participants were volunteers, and no incentives were provided to participants.

This study was conducted with the understanding and written consent of all participants, who had been informed of the research procedures and purposes according to the Declaration of Helsinki, and with approval from the local Ethical Committee (Università degli Studi di Milano; protocol code: 27/19).

After enrollment, participants were assigned to two experimental groups with pair matching method: ULNB and URNB. Ten of them were assigned to the unilateral left nostril breathing group (ULNB: 6 women, 4 men; M_age_ = 22.7; SD = 2), while the other ten were assigned to the unilateral right nostril breathing group (URNB: 6 women, 4 men; M_age_ = 23.2; SD = 2.7). Gender, age, and laterality quotient were the controlled paired variables among the two groups. Considering that data distribution was not normal for all the considered variables across the two groups, and in consideration of the limited sample size, we decided to perform non-parametric analyses to be more prudent and coherent about the analysis plan. No differences were found among groups regarding age, laterality quotient, or personality traits as assessed using non-parametric independent-samples Mann–Whitney U test (all *p* > 0.05; see Supplementary Materials).

### 2.2. Procedure

**Figure 1:**
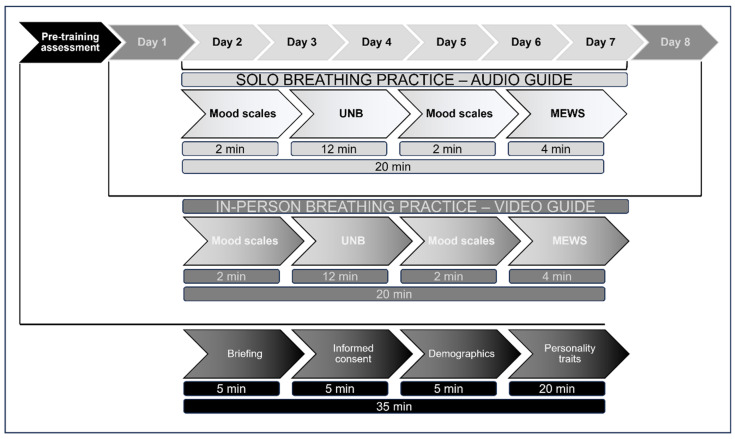
An outline of the experimental procedure. The participants were first briefed, and the inclusion/exclusion criteria were checked (I). Afterward, they read and signed the informed consent forms, provided demographical information and handedness and filled out personality questionnaires (II). Then, the training began (day 1, III). First, they were asked to complete the mood scales. Second, they executed the breathing technique. Third, they completed the mood scales again. Fourth, they completed the MEWS for intrusive occurrences. From day 2 to day 7 (IV), the participants continued with solo training following the same procedure. Finally, (V), the participants concluded the training with the last in-person session, as in phase III.

(I) Participants were asked to complete forms regarding their demographics and general information (age; Edinburgh Inventory: laterality quotient; education) and personality scales (BIG-5; High Sensitivity Trait, HST; Behavioral Inhibition/Activation Scales: BIS/BAS; Mind Wandering Inventory: MWI; Tellegen Absorption Scale: TAS). Personality traits were controlled to ensure a balanced composition of groups (see Supplementary Materials).
(II) Participants were assigned with pair matching method to one of the two experimental groups: ULNB or URNB. Gender, age, and laterality quotient were the controlled paired variables among the two groups.
(III) First trial of in-person breathing training was carried out with pre/post-assessment of mood and post-breathing assessment of mind wandering.
(IV) Six days of solo breathing training (audio-guide) were carried out with pre/post-assessment of mood and post-breathing assessment of mind wandering.
(V) Eighth trial of in-person breathing was carried out as in phase IV (see Figure 1).

The procedure applied was the same for all participants. They were individually seated in a quiet room with the same experimenters. For phase 4, related to the breathing training, the lights were dimmed, and the participants were seated on comfortable chairs to create a relaxing environment.

### 2.3. Instruments

#### 2.3.1. Mood Assessment

**Mood Scales**: Before and after each breathing session, mood was assessed using mood scales. They are useful tools for quickly measuring subjective experiences—in our case, emotional states. Participants were asked to indicate how they are feeling at present by indicating the level of intensity of five emotional states on a scale ranging from 1 (emotion absent) to 10 (emotion very present). The states assessed were stressed, mentally lucid, happy, calm, and restless [43].

#### 2.3.2. Mind-Wandering State Assessment

The **Mind Excessive Wandering Scale** (MEWS) is a self-report scale composed of 15 items that assess mind-wandering traits [44]. Participants were asked to express the frequency with which they experience certain situations using a Likert scale ranging from 0 (never) to 3 (practically always). Participants were asked to express their levels of agreement with each statement on a Likert scale ranging from 0 (never) to 3 (practically always).

For the purposes of this research, we modified the questionnaire so that it would reflect mind-wandering states, i.e., mind wandering experienced during a specific task (breathing training). We therefore selected and adapted 11 items, choosing from those that could be applied to mind-wandering state assessments. Selected items were 1, 2, 3, 5, 7, 8, 11, 12, 13, 14, and 15. They were re-worded to fit the mind-wandering experience. For example, for item 1, the original version (“I have difficulty controlling my thoughts”) was modified with “I had difficulty controlling my thoughts”. We discarded the items that could not be applied to the meditative experience (e.g., “I try to distract myself from my thoughts by doing something else or listening to music”).

Due to the limited sample size, we could not perform a confirmatory factor analysis [45,46,47]. However, to verify the reliability of the new modified scale, Cronbach’s Alpha was calculated for each day, highlighting excellent internal consistency (all α ≥ 0.9).

#### 2.3.3. Pranayama Technique

**Forced unilateral nostril breathing** (UNB) is typically executed by plugging one nostril to allow air to pass through the other nostril. In our protocol, participants were asked to adopt a comfortable and dignified position, with their backs upright and their chins slightly tilted toward the chest, to facilitate breathing movements. After finding a comfortable position, participants were asked to carry out Vishnu Mudra with the dominant hand, which is achieved by lowering the index and middle fingers toward the palm to press the thumb or ring finger on the corresponding nostril. The pressure should be light and applied at the top of the nostril. Since all of our participants were right-handed, the thumb was pressed to close the right nostril, while the ring finger closed the left nostril. This made it possible to regulate respiratory flow and direct it through only one of the two nostrils, both during the inhalation and exhalation phases. In the case that Vishnu Mudra was found to be uncomfortable, another mudra was proposed: Nasagra Mudra. This is achieved by placing the index and middle fingers in the space between the eyebrows and using the thumb and ring finger to close the nostrils as in the previous variation. The non-dominant hand can remain resting on the knee, palm up or palm down.

To ensure a standardized procedure across participants, we created two video tutorials under the supervision of a certified yoga instructor to be watched and followed during the first and eighth in-person sessions, and six audio-guides for the autonomous training, from day 2 to day 7.

**Video tutorials:** Two video tutorials were created: one for the unilateral right nostril breathing (URNB) group, and another for the unilateral left nostril breathing (ULNB) group. They last 4–5 min each and contain all of the practical instructions for performing the training correctly. In addition to detailed instructions for practicing pranayama, the videos provide a visual demonstration of the two techniques, along with some tips for effective and comfortable breathing.

**Audio guides**: Audio guides were designed to accompany practitioners step by step through the six days of autonomous training. The aim was to ensure that all participants performed breathing as correctly as possible, both technically and in terms of the thoughts/attitudes associated with it. We created 6 different versions, one for each day, to avoid the habituation effect. The audio includes both guidance on the correct execution of the practice and elements derived from mindfulness meditation, which include careful observation of the breath and bodily sensations, awareness of one’s own internal states, and focused and non-judgmental attention.

To ensure standardization across training days and conditions, video and audio guides were created by the same person and were identical in length, style, and structure. Furthermore, participants were initially contacted, briefed, and evaluated for inclusion criteria by a researcher who was not involved in the treatment procedures. To avoid placebo and experimenter’s effects, as well as performance or social desirability bias, no information about expected results concerning breathing training was provided to the participants, nor hypotheses about the differential effects of left or right-nostril breathing. Only one researcher was involved in the administration of the training procedures. Also, both the training (pre-recorded standardized video/audio material) and the assessment (online questionnaires) were self-administered to avoid observer, interviewer, or researcher bias.

## 3. Results

Four sets of analyses were performed to answer the research questions:

(I) Descriptive statistics were conducted to explore the trend of the outcome variables (mood and mind wandering) over time.
(II) To investigate the efficacy of the breathing practice, we compared the pre/post-practice scores for the mood scales and the pre/post-training mind-wandering occurrences by conducting the Wilcoxon test.
(III) To assess the extent of the benefits of the practice based on time, we compared the mood and mind wandering scores from day 1 to day 8 by conducting the Friedman test.
(IV) To understand whether one practice was more effective than the other, we compared the outcome scores between the groups by conducting the Mann–Whitney U test for the delta mood scores, and the Friedman test for the mind wandering scores.

### 3.1. Descriptive Statistics

#### 3.1.1. Mood Scales

**Figure 2:**
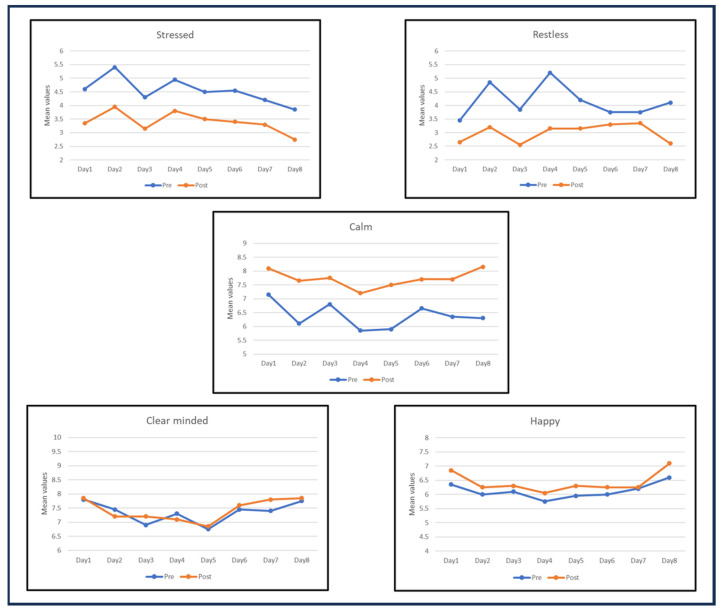
Line charts of mean self-assessed scores for stressed and restless (**up**), calmness (**center**), as well as clear minded and happy moods (**bottom**) for each day before (blue) and after (orange) breathing practice.

In the following graphs, it is possible to visualize the trends of the five moods during the 8 days of training by observing the pre- and post-practice scores (see Figure 2). From a qualitative point of view, perceived stress and restlessness were decreased after the practice, while calmness was improved. For happiness, the trend was similar, but the effect was smaller, while for clarity of mind, the two lines overlapped.

Also, for stressed and restless moods, it is possible to notice how greater improvements were made in the first days of the training program, with a settle in the scores during the second half of the training program.

For calmness, instead, greater improvements occurred as the training progressed. Finally, for some scales, it was possible to notice a difference between in-person and solo practice. In detail, the restlessness, calmness, and happiness scores seemed to benefit more from the presence and guidance of the instructor. These trends are better addressed by the following inferential analyses (from Section 3.2 onward).

#### 3.1.2. Mind Wandering

**Figure 3:**
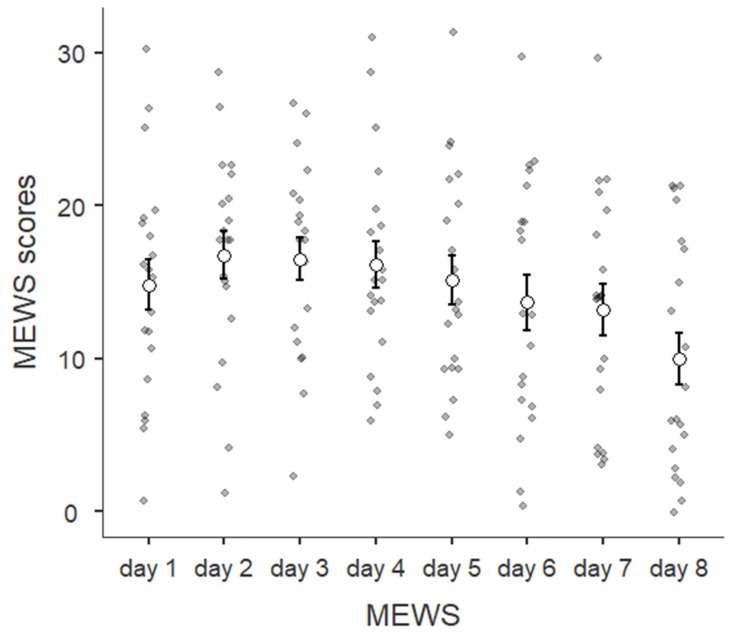
After each daily practice, the participants assessed their experiences with mind-wandering intrusions. In the plot above, the post-practice MEWS scores are represented for each day.

The modified version of the Mind Excessive Wandering Scale was administered only after the practice since it referred to mind fluctuations experienced during the practice. Thus, in the graph, it is possible to visualize the trend during the 8-day training program (see Figure 3).

The trend is, in general, favorable. The participants reported, on average, fewer occurrences of mind wandering over time. However, it is interesting to note that lower scores were registered during training, with the assistance of the video guide, for the mood assessment. Starting from these first descriptive data, we obtained inferential statistics. Considering that data distribution was not normal for some of the outcome variables, we conducted non-parametric analyses for the following steps.

### 3.2. The Efficacy of UNB

To verify the efficacy of the training, we considered the average scores of the mood scales before and after the practice, and we included the pre- and post-training mind wandering values.

#### 3.2.1. The Efficacy of Daily Practice over Mood States

**Figure 4:**
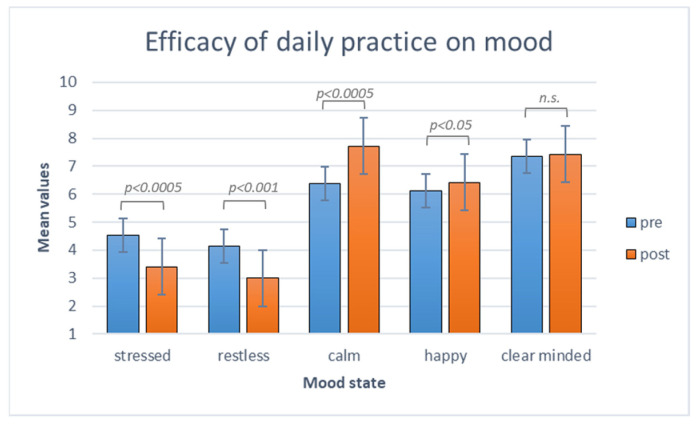
Before and after each daily practice, the participants completed the mood scales about stressed, restless, calm, happy, and clear minded states. In the histogram above, the mean mood states before (blue) and after (orange) the daily practice are represented. Over each comparison, significant and non-significant (n.s.) effects are indicated.

The non-parametric Wilcoxon Test was applied to each mood state between pre- and post-practice values. The analysis returned significant results for stressed (z = −3.73; *p* = 0.00019; r = −0.59) and restless moods (z = −3.41; *p* = 0.00065; r = −0.54), with lower self-assessed scores post-practice training (M_stressed_ = 3.4; sd_stressed_ = 2.1; M_restless_ = 2.99; sd_restless_ = 1.5) than pre-practice training (M_stressed_ = 4.54; sd_stressed_ = 2.17; M_restless_ = 4.14; sd_restless_ = 1.8). Similarly, happy (z = 2.32; *p* = 0.02014; r = 0.37) and calm moods (z = 3.74; *p* = 0.00018; r = 0.59) improved thanks to the practice, with higher self-assessed scores post-practice training (M_happy_ = 6.42; sd_happy_ = 2.05; M_calm_ = 7.72; sd_calm_ = 1.39) than pre-practice training (M_happy_ = 6.12; sd_happy_ = 2.01; M_calm_ = 6.49; sd_calm_ = 1.41). No significant results were found for clarity of mind (see Figure 4).

#### 3.2.2. Mind Wandering

**Figure 5:**
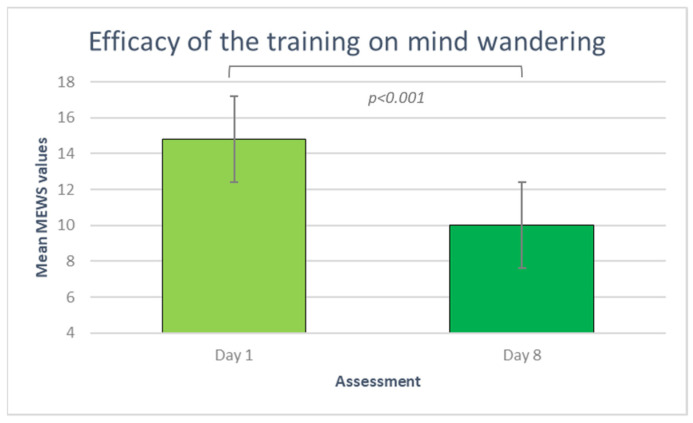
Mean mind wandering scores as recorded by participants on day 1 (light green) and day 8 (dark green) of training.

To verify the efficacy of the pranayama techniques, we conducted a non-parametric Wilcoxon Test on the MEWS scores after the first and eighth practice. The test was significant (z = −2.58; *p* = 0.0099; r = −0.41), with fewer mind-wandering occurrences at the end (M = 10; ds = 7.55) than at the beginning (M = 14.8; ds = 7.43) of the training program (see Figure 5).

### 3.3. Training Progression

To verify the training progression day by day, we calculated the delta values for the mood scores by subtracting the post- and pre-practice values for “happy”, “calm”, and “clear minded” and by subtracting the pre- and post-practice values for “stressed” and “restless”. This way, we obtained a measure of the gains experienced from the practice. Then, we compared the delta values of each day with each other. For mind wandering, the daily scores were compared to each other.

#### 3.3.1. Mood States

We performed a non-parametric Friedman test over the delta values from days 1 to 8 for each mood scale. The Bonferroni correction was applied for multiple comparisons. Although the test returned a significant result for restlessness scores (χ^2^(7,20) = 20.3; *p* = 0.0049; W = 0.15), multiple comparisons did not reach statistical significance. From a qualitative point of view, it is possible to notice a drastic decrease in the gains obtained for self-assessed restlessness after the second half of the training program, specifically from day 4 (M = 2.05; ds = 1.93) to day 7 (M = 0.4; ds = 2.14), indicating that there were larger improvements at the beginning of the training (see Figure 2).

#### 3.3.2. Mind Wandering

**Figure 6:**
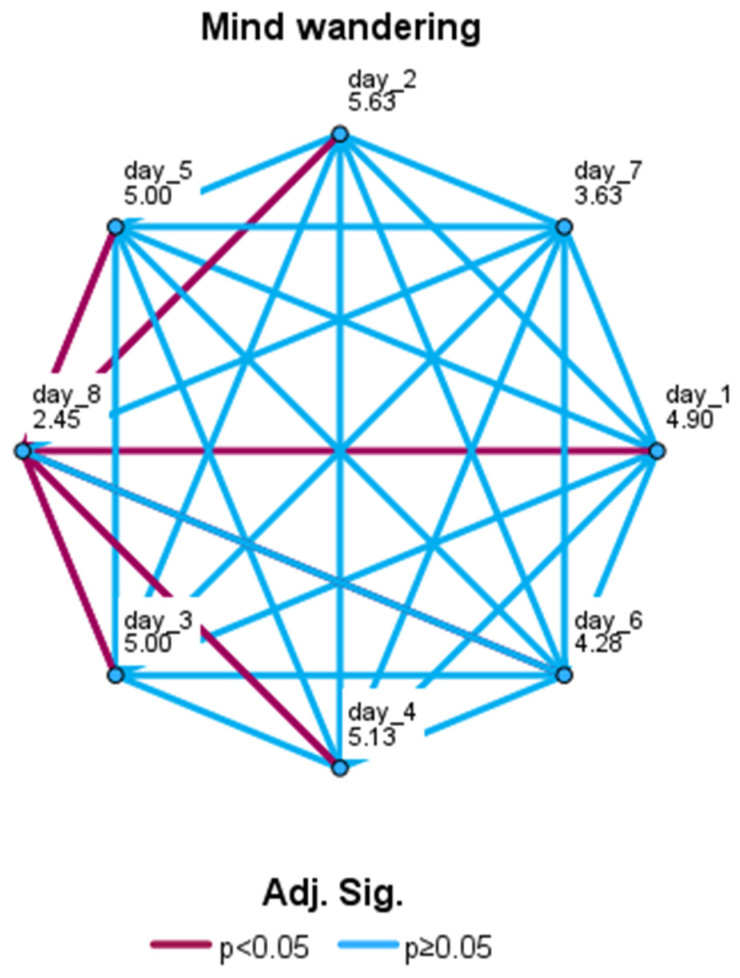
Pairwise comparisons of daily mind wandering values with Friedman test. Significant values were adjusted by Bonferroni correction for multiple comparisons. Each node represents daily assessment and shows average rank. Significant comparisons are displayed in Bordeaux; non-significant comparisons are displayed in blue.

We performed a non-parametric Friedman test for the daily mind wandering values. The Bonferroni correction was applied for multiple comparisons. The test returned a significant result (χ^2^(7,20) = 25.11; *p* = 0.00073; W = 0.18). In detail, the mind-wandering occurrences recorded during the last (eighth) day were significantly lower (M = 10; sd = 7.55) than those assessed at day 1 (*p* = 0.044; M = 14.8; sd = 7.43), day 2 (*p* = 0.0012; M = 16.8; sd = 7), day 3 (*p* = 0.028; M = 16.5; sd = 6.36), day 4 (*p* = 0.016; M = 16.2; sd = 6.82), and day 5 (*p* = 0.028; M = 15.1; sd = 7.1; see Figure 6).

### 3.4. Comparison between Left and Right Nostril Breathing

To investigate the efficacy of one practice over the other, we compared the delta values for each mood across groups. For the mind wandering scale, we subdivided the database and then compared the daily values separately for the two groups.

#### 3.4.1. Mood Scales

**Figure 7:**
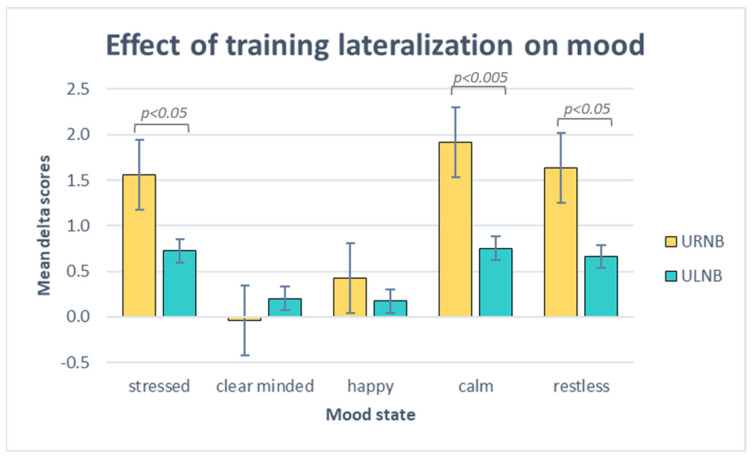
Mean delta values (improvement after practice) for mood states, self-assessed by URNB (yellow) and ULNB (water green) groups.

The Mann–Whitney U test was used for independent samples and identified significant differences between the groups for stressed (z = −2.39; *p* = 0.015; η^2^ = 0.3), restless (z = −2.01; *p* = 0.043; η^2^ = 0.21), and calm moods (z = −2.84; *p* = 0.00288; η^2^ = 0.43), while no significant results emerged for clarity of mind and happiness (see Figure 7).

In detail, the URNB group obtained higher gains than the ULNB group for stressed (M_URNB_ = 1.56; sd_URNB_ = 0.64. M_ULNB_ = 0.73; sd_ULNB_ = 0.66), restless (M_URNB_ = 1.64; sd_URNB_ = 0.9. M_ULNB_ = 0.66; sd_ULNB_ = 0.9), and calm states (M_URNB_ = 1.91; sd_URNB_ = 0.83. M_ULNB_ = 0.75; sd_ULNB_ = 0.64).

#### 3.4.2. Mind Wandering

**Figure 8:**
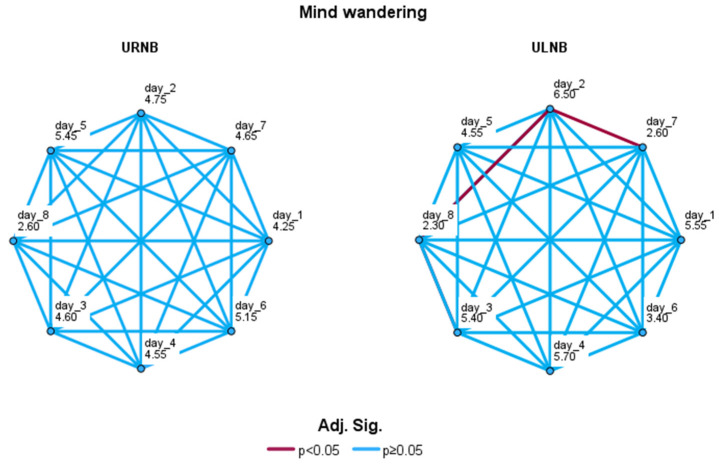
Pairwise comparisons of daily mind wandering values with Friedman test for URNB group (**left**) and ULNB group (**right**). Significance values were adjusted by Bonferroni correction for multiple comparisons. Each node represents daily assessment and shows average rank. Significant comparisons are displayed in Bordeaux; non-significant comparisons are displayed in blue.

We performed a non-parametric Friedman test for the daily mind wandering values separately for the two groups. The Bonferroni correction was applied for multiple comparisons. The test did not return a significant result for the URNB group, while it was significant for the ULNB group (χ^2^(7,10) = 29.12; *p* = 0.00014; W = 0.42; see Figure 8).

**Figure 9:**
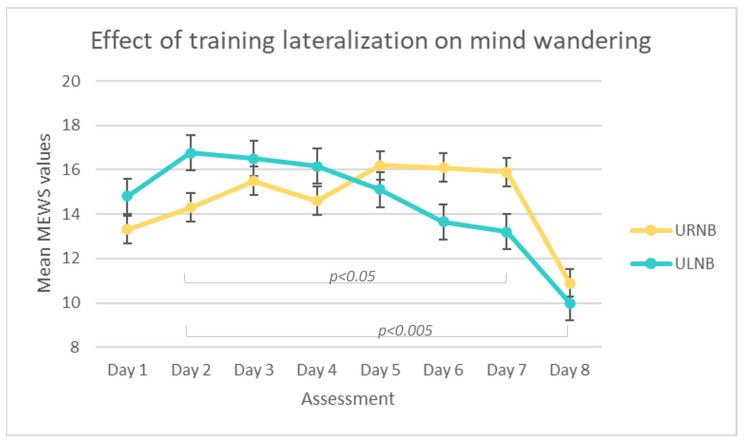
Line charts of mean MEWS scores for each day for URNB (blue) and ULNB (orange) groups.

To understand at what stage the differences are significant, we checked the pairwise comparisons. In detail, the mind-wandering occurrences recorded during day 2 were significantly higher (M = 19.2; sd = 6.23) than those assessed at day 7 (*p* = 0.0104; M = 10.5; sd = 7.09) and day 8 (*p* = 0.00353; M = 9.1; sd = 7.39; see Figure 9).

### 3.5. Summary of Results

Overall, our results show that the proposed meditation training has positive effects on both perceived mood and mind wandering. Considering the time progression, we see that the breathing technique has an immediate effect on positive emotions (e.g., calmness), but it takes longer to see an effect on mind wandering. This is plausible considering that the breath-based meditation method used leads to peripherical physiological changes related to relaxation and stress relief. In contrast, the impact on mind wandering involves a functional reorganization of certain cortical areas, which can only occur after some practice. Specifically, URNB (right nostril) seems to have a markedly greater effect on the various mood parameters measured, while ULNB (left nostril) is shown to have a more active effect on mind wandering with a lower effect on emotions and stress.

## 4. Discussion

The present study was set up and implemented to test the following two hypotheses, which we will discuss separately:

(1) URNB induces an increase in high-arousal emotion (e.g., happiness) and a decrease in mind wandering.

Our data show that URNB had a high impact on psychophysical wellbeing, with decreased perceived stress and restlessness scores, together with increased calmness. These data are, at least partially, innovative since we did not expect to find decreased stress. Since URNB is associated with the activation of the sympathetic system, we expected to find an increase in energizing emotions and clarity of mind instead of calmness and relaxation. However, it is possible that URNB does not have a direct unique interaction with a single hemisphere, but that its effect is due to the modulation of a larger and incompletely lateralized neuro-functional system, calling for a more complex explanation of breathing-related effects [11]. For example, in their classical studies, Block and colleagues [25] found that unilateral forced breathing influenced men’s and women’s cognitive performances differently. In men, it was possible to identify an ipsilateral effect (right nostril -> right hemisphere -> better visuospatial performance; left nostril -> left hemisphere -> better verbal performance), while in women, the effect was partially contralateral (left nostril -> right hemisphere -> better visuo-spatial performance). Due to the sample size, we could not statistically test the gender-related hypothesis, but it could be a significant aspect to consider in future studies.

Furthermore, though traditional research associated UNB with the activation/deactivation of a specific hemisphere, to understand the psychological impact of breathing techniques, it is probably better to associate breathing with the increase or decrease in the activity of specific systems (in particular, the sympathetic vs. the parasympathetic one [14,18]). Even if some research has shown an association between breathing and EEG modulation with animal models [48], specifically within gamma rhythm, further information is still required to understand how this evidence may apply to humans and how physiological changes correlate with emotional and cognitive ones. For example, a previous EEG study by Werntz and colleagues [18] revealed that the oscillations of cerebral hemispheric activity are coupled to the nasal cycle, and different studies showed a contralateral effect [11,12,22]. However, other researchers failed to replicate the effects, finding an ipsilateral [25] or an absent relationship [26,27] instead.

It is possible to hypothesize that the URNB effect was not a direct consequence of the activation of the left hemisphere, but instead, it was probably the result of a bilateral down-modulation of emotion through the synergic work of pre-frontal areas onto the limbic system. Mason and colleagues [29] showed the existence of a set of areas that can be directly modulated by voluntarily controlled breathing, including prefrontal areas (the medial frontal and orbitofrontal cortex, motor, and premotor areas), the insula, and the amygdala. Our results seem to suggest that this modulation is particularly effective through URNB.

In summary, the numerous studies conducted with the variety of effects already described leads us to think that the relationship between unilateral breathing and functional brain organization is complex and calls into question connections on both the left–right and anterior–posterior axes. The resulting complexity calls for more sophisticated analyses aimed at analyzing brain dynamics and its complexity rather than its individual elements.

(2) ULNB increases mind wandering.

Our data strongly suggest that ULNB was more effective in reducing mind-wandering occurrences. As suggested by several studies, while intentional mind wandering may have a positive impact on creativity [49] and other cognitive functions [50], unintentional thinking activity may decrease attention and cognitive performance [51,52]. Thus, since we measured unintentional mind wandering, it is possible to suggest that the effect of ULNB can also be beneficial for cognitive performance, especially when creativity or planning processes are implied.

To better address the modulatory effect of ULNB on cognition, it is important to address its relationship with the parasympathetic effect on the brain. In particular, the synergic functional organization of the prefrontal Executive Control Network (ECN), which is a system that includes the anterior cingulate cortex and the lateral prefrontal cortex [53], and the DMN might be differently affected by sympathetic and parasympathetic activity [54]. Previous studies highlighted a specific association between parasympathetic markers (e.g., cardiac vagal activity), cognitive flexibility, cognitive inhibitory processes, and cognitive conflict management, which are all related to executive control [55,56]. This connection stems from the hypothesis that both parasympathetic activity and executive control are modulated by a top-down pre-frontal system [57]. In detail, parasympathetic and sympathetic activities are differently associated with attention. The Attentional Network Theory [58] posits the existence of an early operating system that modulates the activity of later higher-order control processes. Three phases are described: alerting (preparing the organism to react to stimuli), orientation (allowing the organism to shape a coordinated and adequate response), and executive control. Alerting is associated with sympathetic activity [59], while orientation and executive control are associated with the parasympathetic one due to the modulation of the latter [60]. It seems plausible, then, that ECN is called in by ULNB, while the DMS might be switched off. The DMN, in fact, is linked to internally focused thinking activity and is then deactivated when external tasks must be accomplished [61]. Thus, a mental status that particularly activates the ECN may indirectly affect the DMN, leading to its deactivation and, consequently, to reduced mind wandering.

Generally, our data support the idea that practicing breathing techniques (UNB in our case) may improve mood and reduce stress [33,62]. We found significant decreases in self-perceived stress, restlessness, and mind wandering, along with an increase in positive emotions, regardless of the nostril involved. These results are confirmed by previous studies in the literature focusing both on the general population [32,63] and on clinical conditions [33,64]. For example, Konrad and colleagues [32] engaged university students in a brief mindful breathing training program and found lower stress scores, higher presence scores, higher motivation for the courses, and better moods. Similarly, patients with concussions who were enrolled in a pilot study by Cook and colleagues [33] reported significant decreases in stress, tension, fatigue, and confusion.

## 5. Conclusions

The significant impact of the breathing rhythm on cognitive and affective processing has been recognized since ancient times, giving rise to multiple practices focused on controlling one’s breath to achieve mental clarity and reduce anxiety, stress, and pain [65,66]. Several studies have been conducted to understand this effect, both from physiological and neurological points of view, shedding light on neuro-physiological processes and functional networks. However, the evidence also highlighted the complexity of this issue, showing that it is not possible to straightforwardly associate a certain breathing pattern with a precise psychological effect.

In our study, we obtained mixed results, since improvements in wellbeing are obtained by both URNB and ULNB. However, when comparing the gains of the two types of breathing, it was seen that URNB produced higher effects on positive emotions, while ULNB was more effective in reducing mind wandering. Since both negative emotions and mind wandering have been associated with stress and negative wellbeing [67,68], both breathing techniques can be considered beneficial, but each of them could be used to reach specific purposes both in clinical and non-clinical populations.

Furthermore, mind wandering significantly decreased as a function of time, so practice progression produced better results. Moreover, from a qualitative point of view, the most effective sessions emerged during in-presence training. Taken together, these findings suggest that some results occur as a function of time, while others can be obtained more rapidly. Single sessions may have a high impact on producing positive emotions, especially using the URNB, coherently with other studies using meditation techniques [69]. Deeper and more lasting outputs require more sessions [70]. Reducing mind wandering may require several sessions of ULNB. Furthermore, in-presence training seems to be more effective than solo training. This consideration can be obvious; however, it is important to use mixed methodologies, since self-administered activities are more flexible and might play a vital role in practical applications in contexts where people cannot move due to health or geographical constraints. Thus, it is important to test and validate methods that include remote and/or asynchronous activities. Previous studies on different mediation practices already proved the validity of asynchronous programs (e.g., [71]).

The present study certainly has some limitations that need to be considered. First, since we presented preliminary evidence from a pilot study, it is particularly important not to draw general conclusions from the present study alone. Future research should include larger samples to include individual characteristics that might affect the results. For example, gender, baseline sympathetic/parasympathetic values, and age could be considered in stratifying the sample, thus allowing for a more precise analysis of the results and related mediation factors. Furthermore, the lack of a control group and other methodological aspects, such as the use of single-item measures, can limit the generalization of the data. A controlled trial should be designed and implemented to test our results and suggest a wider use of breathing-based meditation to improve wellbeing in the general population or to complement traditional treatment for specific conditions like depression or anxiety disorders. A more focused study could test the possibility of using our simple but potentially effective training program to help people cope with critical situations, such as severe or chronic clinical conditions. The right nostril breathing technique might be particularly effective in reducing negative emotions and facilitating relaxation and recovery. Finally, interesting future applications of our data could involve the area of learning and attention disorders to help people increase focus and reduce unintentional mind wandering. Previous studies already addressed this issue, often reporting positive results [72], and our data suggest a that more focused approach could also be more effective. Indeed, unilateral left nostril breathing, due to its effect on ECN and DMN, could be particularly effective in facilitating cognitive and learning performance.

Future studies should not only focus on specific populations with more controlled procedures but also include longitudinal tracking of breathing effects. Furthermore, the use of bio-signal recording systems during lab sessions (but also in a home setting), could be informative about the neuro-physiological correlations of breathing and to compare scientific data with the yogic tradition.

In conclusion, despite the limitations of the present study, we think that our results are promising and original. Most previous studies focused on cognition. Here, we had the opportunity to increase awareness of the effects of breathing on cognition and emotions by integrating traditional and scientific views for future applications.

## Data Availability

The data presented in this study are available upon request from the corresponding author. The data are not publicly available due to privacy concerns.

## Supplementary Materials

The following supporting information can be downloaded at https://www.mdpi.com/article/10.3390/brainsci14040302/s1, Table S1: Group matching checked with Mann-Whitney U Test for age and personality variables.
