# Supplementary material for: Breathing Right… or Left! The Effects of Unilateral Nostril Breathing on Psychological and Cognitive Wellbeing: A Pilot Study
Source: Brain Sci. 2024 Mar 23;14(4):302. doi: 10.3390/brainsci14040302 (PMC11048276; doi:10.3390/brainsci14040302)
Supplement: Supplementary file 1 [file brainsci-14-00302-s001.zip › brainsci-2875987-supplementary.pdf]

S. 1

Procedure for groups matching

Controlled variables across groups:

Handedness

Edinburgh Handedness Inventory (EHI). The questionnaire was developed to objectively assess which hand is normally used during certain everyday activities [39], of which we used the modified, shorter version [40]. Participants are asked to specify, for each item, which hand they usually use. If the preference is for a certain side, a (+) is assigned in the corresponding column. If the preference is so strong that the other side is never used, (++) is attributed. If either side is used indifferently, a (+) is assigned to both hands. The final score is called the "Laterality Quotient" and is calculated using the following formula: L.Q. = (R-L)/(R+L)*100, where R is the number of (+) assigned to the right side and L is the number of (+) assigned to the left side.

Personality Measures

BIG-5 Personality Questionnaire: For the present paper we used the Big Five Inventory-10 (BFI-10) [41], which consists of 10 items to be rated on a five-step scale from 1 ("disagree strongly") to 5 ("agree strongly"). It explores the Big Five Factor Model that includes the factors Extraversion, Agreeableness or Friendliness, Conscientiousness, Emotional Stability or Neuroticism, and Intellect or Openness to Experience.

Behavioral Inhibition/Activation Scales (BIS/BAS). The scale includes 24 items (20 score items and four fillers) to be rated on a five-point Likert scale from 1 "not at all true" to 5 "completely true" [42,43]. The scoring included two subscales, one for BIS (7 items), and three for BAS (BAS-reward responsiveness; BAS-drive; BAS-fun seeking: 13 items).

Highly Sensitive Person Scale (HSPS) [44]: Aron and Aron suggested that people could differ in the way they neurologically transmit and process sensory information [45]. It is the case of sensory processing sensitivity (SPS), described as a personality trait modulated by genetic factors, which allows people to feel and process more information at one time, and in a deeper way. Such sensitivity is referred to both external and internal stimuli [46]. Accordingly, Highly Sensitive Persons are more inclined to experience higher arousal during exposure to environmental stimuli such as bright lights, strong smells, and noisy and chaotic situations. To measure SPS Aron & Aron [44] implemented the HSPS, which includes 27 statements towards which the participant has to express their degree of agreement on a scale ranging from 1 ("totally disagree") to 7 ("totally agree").

Mind Wandering Inventory (MWI): The MWI is a recently developed instrument [47] to measure trait-mind-wandering as experienced in everyday life. The questionnaire consists of 10 items assessed via a Likert scale ranging from 1 (completely agree) to 4 (completely disagree). The first 3 items refer to detachment from one's surroundings; the following 3 items measure the tendency of the mind to wander in space and time; the last 4 capture variation in mental content and the spontaneous flow of thoughts.

Tellegen Absorption Scale (TAS): Absorption is a personality trait that reflects an individual's cognitive capacity to become fully engaged with sensory and imaginative experiences, to the extent that perception, memory, behavior, and emotional state are affected [48]. Absorption was measured with the original version of the TAS [49]. It is a questionnaire consisting of 34 items, which assess

openness to experience, one's level of emotional and cognitive involvement in a range of situations, disposition to experience altered states of consciousness, and the quality of thoughts and memories. Participants were asked to tick only those items that correspond to their experience. The number of marked statements reflects the individual's level of absorption.

Table S1. Mann-Whitney U Test for age and personality variables between groups.

VARIABLE	MANN-WHITNEY U	STANDARD ERROR	STANDARDIZED TEST STATISTICS	ASYMP. SIG. (2-TAILED)	EXACT SIG. [2*(1-TAILED SIG.)]
Age	48	13.05	-.15	.88	.91
Laterality quotient	53.5	12.61	.28	.78	.8
Extraversion	48	13.04	-.15	.88	.91
Agreeableness	59	12.98	.69	.49	.53
Conscientiousness	47	12.75	-.24	.81	.85
Emotional stability	41	13.06	-.69	.49	.53
Openness	34.5	12.97	-1.2	.23	.25
High Sensitive Trait	49	13.22	-.08	.94	.97
BIS	65.5	13.16	1.18	.24	.25
BAS-D	35.5	13.12	-1.11	.27	.28
BAS-FS	42	13.08	-.61	.54	.58
BAS-RR	40	13.08	-.77	.45	.48
Mind wandering	62.5	13.19	.95	.34	.35
Absorption	37.5	13.13	-.95	.34	.35
